# Cecal volvulus in Marfan Syndrome

**DOI:** 10.1093/jscr/rjac543

**Published:** 2024-11-22

**Authors:** Swee Yan Yip, Amir Rashid, Stephen Ward

**Affiliations:** Department of General Surgery, Queen Elizabeth Hospital Birmingham, University Hospital Birmingham NHS Trust, Birmingham B15 2GW, United Kingdom; Department of Radiology, Queen Elizabeth Hospital Birmingham, University Hospital Birmingham NHS Trust, Birmingham B15 2GW, United Kingdom; Consultant Surgeon & Senior Research Fellow, University of Birmingham, Vincent Drive, Birmingham B15 2TT, United Kingdom

**Keywords:** Marfan, volvulus, colorectal, surgery

## Abstract

Gastrointestinal pathology in adult patients with Marfan Syndrome is rarely reported in literature. Nevertheless, it could be life threatening when it occurs. In our paper, we are presenting the first reported case of caecal volvulus in an adult patient with Marfan Syndrome, our findings and management. We also discuss the more common radiological findings that may enhance decision making amongst surgical clinicians. A high index of suspicion and a multidisciplinary approach is advised when encountering these group of patients.

## Introduction

Gastrointestinal pathology in adult patients with Marfan Syndrome is rare. Seldom, it is described in the paediatric population. Patients with Marfan Syndrome are primarily diagnosed with skeletal, ocular and cardiovascular complications. Here, we present the first reported case of caecal volvulus in an adult patient with Marfan Syndrome, our findings and management.

## Case report

**Figure 1:**
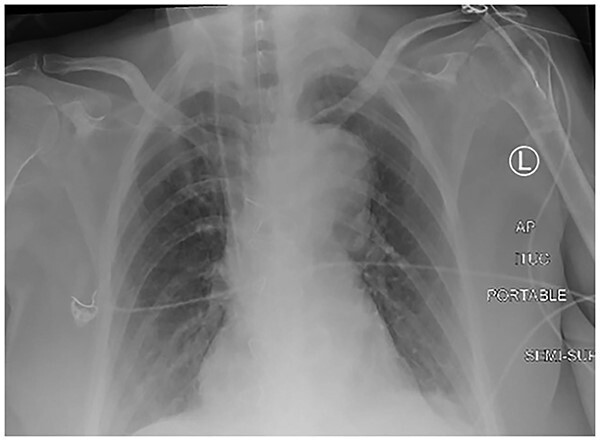
Erect chest X-ray demonstrating left pleural effusion and an unfolded, dilated aortic arch.

**Figure 2:**
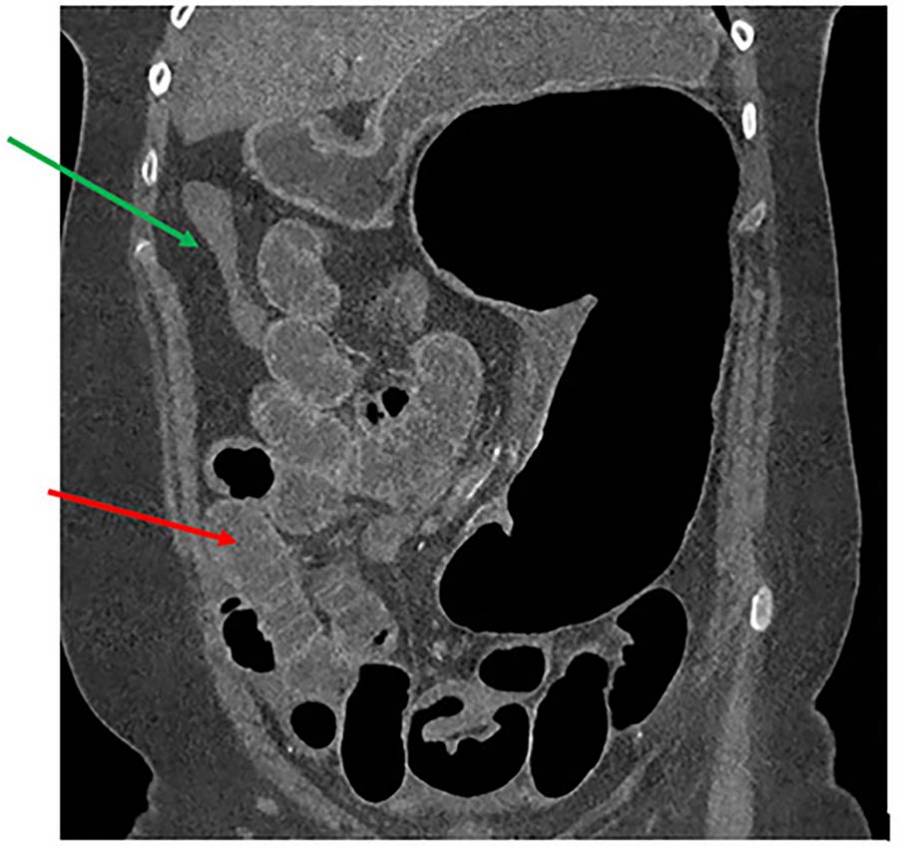
CT thorax, abdomen and pelvis coronal view demonstrating a distended, twisted caecum, caecum in the left upper quadrant, small bowel distension (red arrow) and a decompressed ascending colon (green arrow).

**Figure 3:**
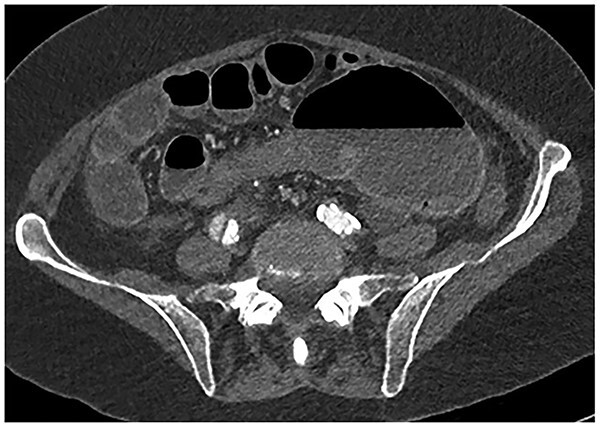
CT abdomen axial view demonstrating the Beaking sign—a progressive tapering of afferent and efferent limbs leading into the twist.

**Figure 4:**
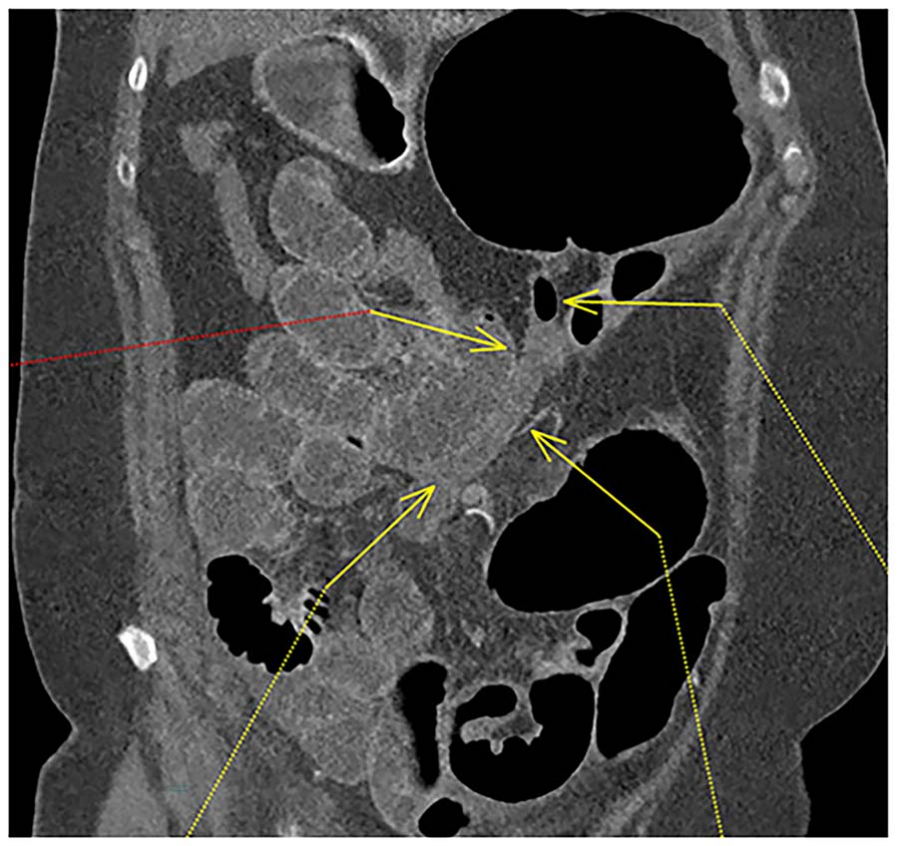
Fluid-filled mid to distal appendix with some gas in the proximal part outlined with yellow arrows. Appendix is seen within the upper abdomen, indicating an increased likelihood of caecal volvulus.

A 61-year-old female with known Marfan Syndrome was admitted to the emergency department with 3-day history of abdominal pain, nausea, vomiting and bowels not opening. Past medical history included three previous episodes of Type A aortic dissection, recent aortic root and ascending aorta replacement alongside bioprosthetic aortic valve replacement. She was an ex-smoker and usually independent at home. On presentation, the patient was haemodynamically stable and apyrexial. Biochemical results were unremarkable except for a Lactate dehydrogenase (LDH) of 309 and a C-reactive protein (CRP) of 101. On examination, she had a diffusely tender and distended abdomen, without signs of peritonism. An erect chest X-ray shows left pleural effusion with a dilated aortic root (Fig. 1). A computed tomography (CT) of thorax, abdomen and pelvis was requested, which demonstrated a caecal volvulus with a dilated cecum of 8.8 cm and upstream small bowel dilatation with air fluid levels, and a chronic aortic dissection extending from the ascending thoracic aorta to the common iliac (Figs 2–4). Following consultation between radiologists, cardiothoracic surgeons and general surgeons, she underwent an emergency laparotomy and right hemicolectomy with side-to-side ileocolic anastomosis. Intraoperatively, the caecal volvulus, comprising a dilated ascending colon measuring up to 10 cm, was delivered and resected along with its mesentery. The patient was admitted to the intensive care unit post-operatively for close blood pressure monitoring and control and stepped down to level 3 wards on day-2 post-operation. Her operation was complicated by a small infected wound haematoma thatwas managed with a 5-day course of ciprofloxacin. A CT of the abdomen and pelvis to investigate a rising CRP on day-9 revealed a subcapsular liver haematoma thath was managed conservatively. The patient was discharged 12-days post-operatively. Histology from the resected colon showed evidence of ischaemia in keeping with a closed loop obstruction such as caecal volvulus. There was also an incidental T1 N0 colonic tumour arising from a sessile serrated polyp and two other serrated polyps. A colonoscopy to inspect the remaining colon has been arranged.

## Discussion

Marfan Syndrome (MFS) is an autosomal dominant connective tissue disorder affecting the fibrillin-1 gene. It affects 1 in 3300 to 3 in 10 000 people. It leads to various skeletal, ocular and cardiovascular complications. Gastrointestinal pathology in the adult population is very rare. The reported gastrointestinal pathologies leading to bowel obstruction in the adult MFS population includes malrotation, volvulus, congenital band, diverticulosis, abnormal aneurysm of the vasculature and hernia [1]. Irritable bowel syndrome was also found to be more common in patients with MFS as compared to patients without MFS [2].

Volvulus is the twisting of loop of intestine around its mesenteric attachments resulting in a closed loop bowel obstruction. If untreated, it will lead to bowel necrosis, bowel ischaemia and consequently death. The incidence of volvulus in the normal population is 2.8–7.1 million people per year [3] and contributes to 1–3% of all large bowel obstructions [4].

Rokitanksky first described caecal volvulus in 1837 [3]. Caecal volvulus is the second most common type of volvulus and has a lower incidence rate than sigmoid volvulus, accounting for only 10% of all intestinal volvulus [3, 5]. It is the torsion of a free and mobile cecum, ascending colon and terminal ileum around their mesenteric pedicles. Cecal volvulus can be secondary to either a developmental failure of peritoneal fixation [4], or from restriction of bowel at fixed point such as adhesions, abdominal mass (pregnancy, constipation), or scarring from calcified lymph nodes within the abdomen acting as a fulcrum for rotation [3, 5]. In hospitalized patients, the development of caecal volvulus is associated with intestinal dysmotility and colonic distension.

Risk factors associated with the occurrence of caecal volvulus are more so from anatomical variations of the gastrointestinal tract rather than ‘acquired’ causes of those of sigmoid volvulus.

Clinical presentation of patients with intestinal volvulus are classically of intestinal obstruction including abdominal pain, constipation, nausea and vomiting [6]. The differential diagnoses are constipation, pseudo-obstruction, acute mesenteric ischaemia, bowel obstruction, diverticular disease and strangulated hernia.

Common findings on radiological imaging are include caecal dilatation, ceacal apex in the left upper quadrant (LUQ), small bowel dilatation, absence of gas in distal colon and single air-fluid level, all which are present in our patient’s imaging (Table 1) [7–10].

**Table 1:** Listing the specificity and sensitivity of radiological signs in cecal volvulus

	Sensitivity	Specificity
Severe cecal distension	45%	100%
Small bowel distention	82%	58%
Distal colon decompression	91%	92%
Caecal apex in the LUQ	36%	100%
Whirl sign	73%	100%

In our patient, we can also appreciate the Beaking sign as demonstrated in our CT abdomen (Fig. 2). Other CT findings described in literature that are not seen in our patient includes Whirl sign – a tightly twisted mesenteric vessel and colon.

To date, there are only a few reported cases of intestinal volvulus in adult patients with MFS [11–15] and a one reported case in children [16]. To our knowledge, this is the first reported case of caecal volvulus in an adult patient with MFS. The outcomes are poor for those who have a delayed diagnosis and hence highlights the importance of early recognition and management.

This case reinforces the need for multidisciplinary consults (clinical, radiological, surgical) in patients with MFS as they can present with a myriad of clinical emergencies such as an aortic dissection and bowel obstruction in our patient. Clinicians should maintain a high index of suspicion in these patients as a prompt diagnosis is deemed lifesaving.

Furthermore, our patient was found to have an incidental early colorectal cancer and polyps. There is convincing evidence of increased risk of malignancy in MFS patients [17], which may be mediated through mutations in the TGFBR2 gene [18]. This study proposes that clinicians should consider additional cancer surveillance for patients with MFS.

In conclusion, intestinal volvulus in adult patients with MS is rare and likely no more common than in the general adult population. Nevertheless, it can be life-threatening if it does occur. Clinicians should maintain a high index of suspicion of gastrointestinal pathologies in patients with MFS diagnosed with abdominal pain and a multidisciplinary approach including cardiology and cardiothoracic surgery may be required. There is an association between MFS and colorectal cancer, although the strength of this association and repercussions in terms of surveillance are presently unclear.
